# Association between CT-Based Preoperative Sarcopenia and Outcomes in Patients That Underwent Liver Resections

**DOI:** 10.3390/cancers14010261

**Published:** 2022-01-05

**Authors:** David Martin, Yaël Maeder, Kosuke Kobayashi, Michael Schneider, Joachim Koerfer, Emmanuel Melloul, Nermin Halkic, Martin Hübner, Nicolas Demartines, Fabio Becce, Emilie Uldry

**Affiliations:** 1 Department of Visceral Surgery, Lausanne University Hospital CHUV, University of Lausanne, 1011 Lausanne, Switzerland; david.martin@chuv.ch (D.M.); michael.schneider@chuv.ch (M.S.); emmanuel.melloul@chuv.ch (E.M.); nermin.halkic@chuv.ch (N.H.); martin.hubner@chuv.ch (M.H.); emilie.uldry@chuv.ch (E.U.); 2 Department of Diagnostic and Interventional Radiology, Lausanne University Hospital CHUV, University of Lausanne, 1011 Lausanne, Switzerland; yael.maeder@chuv.ch (Y.M.); joachim.koerfer@epfl.ch (J.K.); fabio.becce@chuv.ch (F.B.); 3 Hepato-Pancreatico-Biliary Surgery Division, Department of Surgery, Graduate School of Medicine, University of Tokyo, Tokyo 113-8654, Japan; kosuke_63@hotmail.com

**Keywords:** liver resection, sarcopenia, computed tomography, outcomes, complications, survival

## Abstract

**Simple Summary:**

Cancer cachexia often includes sarcopenia, which is characterized by a progressive, generalized loss of skeletal muscle mass and strength, combined with fatty infiltration into the muscle. Sarcopenia has been considered a patient-specific imaging biomarker for predicting outcomes after cancer surgery. The present study aimed to evaluate whether preoperative sarcopenia was associated with postoperative outcomes and survival in patients that underwent liver resections. Sarcopenia, assessed by preoperative CT imaging, was present in two-thirds of patients. Independent risk factors for sarcopenia were age, male sex, ASA score ≥ 3, and malignancies. Based on CT assessment alone, sarcopenia had no impact on clinical outcomes or overall survival after hepatectomy.

**Abstract:**

This retrospective observational study aimed to evaluate whether preoperative sarcopenia, assessed by CT imaging, was associated with postoperative clinical outcomes and overall survival in patients that underwent liver resections. Patients operated on between January 2014 and February 2020 were included. The skeletal muscle index (SMI) was measured at the level of the third lumbar vertebra on preoperative CT scans. Preoperative sarcopenia was defined based on pre-established SMI cut-off values. The outcomes were postoperative morbidity, length of hospital stay (LOS), and overall survival. Among 355 patients, 212 (59.7%) had preoperative sarcopenia. Patients with sarcopenia were significantly older (63.5 years) and had significantly lower BMIs (23.9 kg/m^2^) than patients without sarcopenia (59.3 years, *p* < 0.01, and 27.7 kg/m^2^, *p* < 0.01, respectively). There was no difference in LOS (8 vs. 8 days, *p* = 0.75), and the major complication rates were comparable between the two groups (11.2% vs. 11.3%, *p* = 1.00). The median overall survival times were comparable between patients with sarcopenia and those without sarcopenia (15 vs. 16 months, *p* = 0.87). Based on CT assessment alone, preoperative sarcopenia appeared to have no impact on postoperative clinical outcomes or overall survival in patients that underwent liver resections. Future efforts should also consider muscle strength and physical performance, in addition to imaging, for preoperative risk stratification.

## 1. Introduction

Older individuals will comprise about one-quarter of the world’s population by 2050. Several studies have shown that increasing age was associated with increased risks of postoperative complications, mortality, and prolonged hospital stays after abdominal cancer surgery [1,2]. The aging process often includes sarcopenia, which is characterized by a progressive, generalized loss of skeletal muscle mass and strength [3,4], accompanied by the infiltration of fat and fibrotic connective tissue into muscle [5]. The latter is the tissue that supports, binds, or distinguishes different types of tissues and organs and is made up of cells and extracellular matrix, which itself is made up of fibers (collagen, reticulin, fibronectin, or elastin) and ground substance [6]. Sarcopenia also occurs in other conditions, including cancer, reduced caloric intake, poor blood flow to the muscles, mitochondrial dysfunction, and a decline in anabolic hormones [7,8,9,10]. A meta-analysis of data on patients that underwent gastrointestinal cancer surgery showed that the prevalence of sarcopenia varied between 12% and 78%, and it was associated with an elevated risk of major and overall postoperative complications [11]. In liver surgery, despite significant improvements in perioperative care and surgical techniques, the morbidity rate has ranged from 20% to 30% [12,13,14]. Moreover, in patients that require liver resections for primary malignancies or colorectal liver metastases, preoperative sarcopenia may predict postoperative clinical and oncological outcomes [15,16,17,18]. Thus, sarcopenia has been considered a patient-specific imaging biomarker for predicting clinical outcomes [19].

A wide variety of tests and tools are available to assess sarcopenia in clinical practice and research, such as dual-energy x-ray absorptiometry and bioimpedance analysis. A validated approach is to measure the cross-sectional areas of specific muscle groups in specific body locations based on imaging. This approach is recommended by the European consensus on the definition and diagnosis of sarcopenia (EWGSOP2) [20]. In addition to muscle cross-sectional areas or volumes representing quantity, muscle quality can also be measured with CT images through its density and fat infiltration [21,22]. However, current studies are limited by relatively small patient cohorts, data heterogeneity, and the lack of a standardized assessment of body composition, including inconsistent measures of muscle quantity and muscle quality and fat content. Frequently, a single sarcopenia parameter is measured, and the methods vary. It would thus be interesting to measure different parameters of body composition with automated methods.

The present study aimed to evaluate whether muscle quantity, muscle quality, and muscle fat content, measured with a semi-automated deep-learning-based method on CT imaging, were associated with postoperative clinical outcomes and overall survival in patients that underwent liver resections.

## 2. Materials and Methods

### 2.1. Study Design and Patients

This single-center retrospective observational study included all consecutive patients that underwent liver resections at the Department of Visceral Surgery, University Hospital CHUV, Lausanne, Switzerland, between 1 January 2014, and 1 March 2020. Patients that underwent resections of another organ during the same surgical procedure were excluded. For all patients, treatment decisions were discussed in a multidisciplinary meeting involving hepatologists, medical oncologists, radiologists, pathologists, and surgeons. Major liver resections were defined as the removal of ≥3 Couinaud’s segments. Preoperative assessments included biological, volumetric, and functional liver parameters. When, according to a decision algorithm, the preoperative liver volume was deemed insufficient, portal vein embolization was performed to increase the size of the liver remnant [23]. In addition, all patients were managed according to the Enhanced Recovery After Surgery (ERAS) protocol [24]. Patients at risk (weight loss > 10–15% within 6 months, BMI < 18.5 kg/m^2^, nutritional risk screenings (NRS) ≥ 3 [25], or serum albumin < 30 g/L) received oral nutritional supplements for 7 days prior to surgery. For severely malnourished patients (>10% weight loss), surgery was postponed for at least 2 weeks to improve nutritional status and allow patients to gain weight. There was no intervention on preoperative physical activity and mobility.

Patient demographics, diagnosis, malignancy, and intraoperative characteristics were collected. The postoperative outcomes included morbidity, mortality, reoperations, and the length of hospital stay (LOS). Complications that occurred within 30 postoperative days were graded according to the Clavien-Dindo classification system [26]. Major complications were defined as grades ≥ IIIb. When a patient developed more than one complication, only the highest grade was retained. Postoperative liver failure and bile leakage were defined according to the International Study Group for Liver Surgery (ISLGS) [27,28].

### 2.2. Sarcopenia Assessment

**Figure 1:**
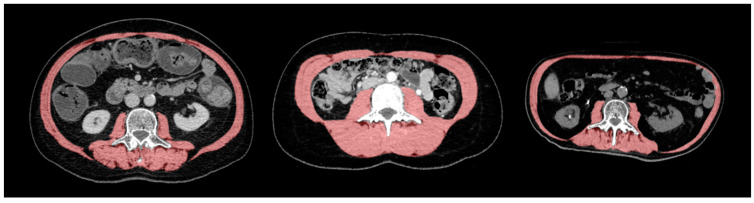
Skeletal muscle area morphology. Computed tomography scans show different morphotypes of skeletal muscle area (SMA) at the third lumbar vertebra (L3) level. This region contains the psoas muscle, paraspinal muscles (erector spinae, quadratus lumborum), and abdominal wall muscles (transversus abdominus, external and internal obliques, rectus abdominus).

Over the study period, preoperative CT scans were performed with different CT systems from various manufacturers, with or without intravenous contrast administration. All CT scans were performed within a maximum time interval of 1 month prior to the intervention and included an abdominal series obtained at a tube potential of 120 kVp. Other data acquisition and image reconstruction parameters varied slightly between patients, inherent to the study design and patient referral for liver surgery. Muscle mass and quality were measured on axial CT slices at the mid-pedicle level of the third lumbar vertebra using a semi-automated, deep-learning-based method with a U-Net architecture algorithm [29,30,31]. Similar methods have recently been validated on large data sets [32,33]. However, because the accuracy, precision, and reliability of the predicted image segmentations cannot be fully guaranteed and trusted by such methods, all automated muscle segmentations were secondarily reviewed and corrected by two board-certified musculoskeletal radiologists successively (with 7 and 16 years of experience) using a custom graphical user interface. To estimate muscle mass or quantity, skeletal muscle area (SMA) was measured in cm^2^ and was normalized by the squared patient height to obtain the skeletal muscle index (SMI, cm^2^/m^2^). Automated muscle segmentations included the psoas muscle, the paravertebral muscles, and the muscles of the abdominal wall (Figure 1). To estimate muscle quality, the skeletal muscle radiation attenuation (SMRA) was measured in Hounsfield units (HU) and based on muscle density. Any given skeletal muscle displays radiation attenuation between −190 and +150 HU [22]. As an additional indicator of muscle degeneration, the intramuscular adipose tissue (IMAT) was determined by measuring the fat pixels within the SMA. CT scans can clearly discern fat from muscle because fat displays negative attenuation values, whereas attenuation for muscle is positive, and attenuation is sensitive to proton content per unit mass, which is high in adipose tissue [34]. The IMAT was also normalized by the squared patient height to obtain the IMAT index (IMATI, cm^2^/m^2^), as previously described [35]. The SMI was used to define sarcopenia, with cut-off values at 52.4 cm^2^/m^2^ for men and 38.5 cm^2^/m^2^ for women, as previously described [36,37,38,39]. Outcomes were compared between patients with and without sarcopenia, as defined by these sex-specific cut-off values.

### 2.3. Statistical Analysis

Continuous variables are expressed as mean (standard deviation, SD) or median (interquartile range, IQR) and compared with Mann-Whitney U test or Student’s *t*-test according to their distribution (Shapiro-Wilk test). Categorical variables are expressed as the frequency and percentage and compared between groups with Pearson’s chi-square or Fisher’s exact test, where appropriate. Correlations between continuous variables were assessed with Pearson correlation coefficients. Logistic binary regression was used for predictive factors of sarcopenia and major complications. Overall survival for patients with malignancies was analyzed with the Kaplan-Meier method, and groups were compared with the log-rank test. Survival was defined as the time interval between the day of the index operation and the date of death due to any cause. All analyses were performed with SPSS 26.0 (SPSS Inc., Chicago, IL, USA).

## 3. Results

**Figure 2:**
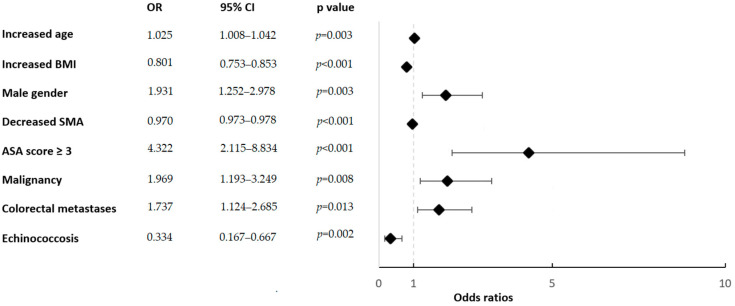
Multivariable analysis results indicate risk factors for sarcopenia. The analysis included all factors identified as significant in univariable analysis (*p* < 0.05). OR = odds ratio; BMI = body mass index; SMA = skeletal muscle area, ASA = American Society of Anesthesiologists. Dotted vertical line: null effect (OR = 1).

Among the 355 patients included, 212 (59.7%) had preoperative sarcopenia, based on the CT analysis and cut-off values used. Patient demographics and surgical details are reported in Table 1. Patients with sarcopenia were significantly older (63.5 years) and had a significantly lower mean body mass index (BMI; 23.9 kg/m^2^) than patients without sarcopenia (mean age: 59.3 years, *p* < 0.01, and mean BMI: 27.7 kg/m^2^, *p* < 0.01). Compared to the group without sarcopenia, the sarcopenia group included significantly more men (65.6% vs. 49.9%, *p* < 0.01), and a larger proportion had American Society of Anesthesiologists (ASA) scores ≥ 3 (24.5% vs. 7.0%, *p* < 0.01). There were no differences between the two groups in terms of comorbidities or surgical procedures, but a larger proportion of patients with sarcopenia had malignancies (82.5%) compared to patients without sarcopenia (69.9%, *p* < 0.01). The risk factors for sarcopenia identified in the multivariate analysis are presented in Figure 2.

The overall complication rates were 46.2% (*n* = 98) in the sarcopenia group vs. 48.3% (*n* = 69) in the group without sarcopenia (*p* = 0.745). A total of 24 patients (11.3%) in the sarcopenia group had major complications vs. 16 patients (11.3%) in the group without sarcopenia (*p* = 1.00, Table 2). For complications specific to liver resection, the groups displayed no significant difference in the rate of bile leakage or postoperative liver failure. There was no 30-day mortality for the entire cohort. None of the sarcopenia indices was a predictive factor of major complications (Table 3). The only significant predictive factor of major complications was the presence of cholangiocarcinoma (OR 2.762, 95% CI 1.218–6.264, *p* = 0.015). A subgroup analysis for patients with major resections did not show any significant differences in terms of postoperative clinical outcomes in patients with or without sarcopenia (Table S1).

The LOS was not significantly different between groups (sarcopenia group: median 8 days, IQR 6–14; no sarcopenia group: median 8 days, IQR 6–16; *p* = 0.753). A very weak, negative correlation was observed between the SMA and the LOS (*r* = −0.110, *p* = 0.038). No correlation was found between the LOS and the SMI (*r* = −0.092, *p* = 0.084), the SMRA (*r* = −0.035, *p* = 0.513), and the IMATI (*r* = 0.003, *p* = 0.961), respectively.

**Figure 3:**
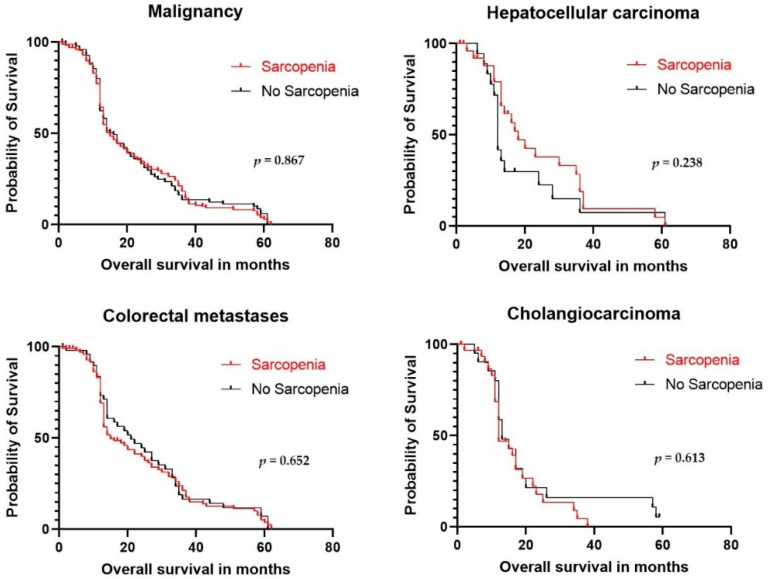
Overall survival for malignancies after hepatectomy. Results are stratified by the presence of sarcopenia, assessed with the skeletal muscle index (SMI).

The overall median survival of patients with malignancies was 15 months in the presence of sarcopenia compared to 16 months for patients without sarcopenia (*p* = 0.867, Figure 3). There was no significant difference in survival for the malignancy subtypes (hepatocellular carcinoma, colorectal metastases, and cholangiocarcinoma).

## 4. Discussion

This study showed that about two-thirds of patients undergoing liver resection had preoperative sarcopenia. The following risk factors for sarcopenia were identified: older age, male sex, high ASA scores, and malignancies. However, no associations were observed between preoperative sarcopenia and postoperative clinical outcomes or overall survival.

Previous studies in gastrointestinal cancer surgery reported a sarcopenia incidence of approximately 50%, based on predefined cut-off values. Moreover, previous studies that used population-tailored cut-offs reported an even lower incidence of 35% [11,40]. In general, CT is routinely performed prior to hepatectomy. Thus, CT scans are considered convenient for assessing sarcopenia without the need for additional tests. However, currently, the cut-off values for identifying sarcopenia in CT are not precisely defined, either for muscle quantity or for muscle quality. In the present study, a pre-established SMI cut-off based on previous studies was used to identify sarcopenia, but no association with the outcomes was found. The SMI is derived from the SMA, and both are muscle quantity indices, while SMRA and IMAT are muscle quality indices. This could explain the fact that there was no difference in SMRA and IMAT between the two groups compared in this study. A hypothesis could be that the quantity and the quality of the muscle do not necessarily match, as already suggested by other studies [31,41,42]. These results contrasted with findings from a recent meta-analysis, where various CT-based sarcopenia indices were evaluated as predictors for the risk of major complications in patients undergoing hepato-pancreato-biliary surgery [43]. That study showed that all the commonly used indices, including SMA, SMI, and SMRA, could predict the risk of major postoperative complications; however, a consensus on cut-offs to define sarcopenia was still lacking.

Independent risk factors for sarcopenia were older age, male sex, ASA scores ≥ 3, and malignancies. In a previous prospective study that assessed associations between sarcopenia and outcomes after liver resections, age and BMI were not correlated with reduced muscle mass or strength [40]. Two other retrospective studies on patients that underwent hepatectomies found that patients with sarcopenia had significantly lower BMIs compared to patients without sarcopenia, but age was comparable between groups [44,45]. Proper body size adjustment for muscle quantity is debated, and sarcopenia in obesity is currently not well described [46]. Age-related decreases in skeletal muscle mass that occur concurrently with obesity, termed “sarcopenic obesity”, might result in a substantially increased risk of morbidity and functional decline [47]. Sarcopenic obesity is the combination of low muscle and high fat mass [48]. Obesity, resulting from an increase in adipose tissue, is also considered a critical cause of skeletal muscle loss that leads to a cycle of continuous fat gain [49]. This could not be demonstrated in this present study, as sarcopenic patients had significantly lower BMI. One hypothesis is that a majority of patients were suffering from malignant pathologies, thus causing cancer cachexia, with loss of fat-free and fat mass, resulting in an overall weight loss (kg). It is estimated that half of all patients with cancer eventually develop cachexia, with anorexia and a progressive loss of adipose tissue and skeletal muscle mass [50]. This syndrome is characterized by systemic inflammation, negative protein and energy balance, and an involuntary loss of lean body mass [51].

Previous studies showed that female sex was a risk factor for sarcopenia [17,18,40,45]. Various endogenous and exogenous factors influence the prevalence of sarcopenia; indeed, such as hormonal changes that enhance the loss of muscle mass and occur more slowly in men than in women [52]. Some studies conducted in the general population reported that the relative reduction in muscle mass was greater in men than in women [53,54]. Sex steroids influence the maintenance and growth of muscles, and decline in androgens, estrogens, and progesterone by aging leads to sarcopenia [55]. These steroid hormones can interact with different signaling pathways through their receptors. However, sex steroid hormone receptors and their exact roles are not completely defined in muscles, and the evidence for an association between sex and sarcopenia remains inconsistent and unclear.

The literature has also shown inconsistent results on surgical outcomes. Several retrospective series have shown that the LOS was prolonged after liver surgery in patients with preoperative sarcopenia [16,17,39,44]. However, two previous studies showed that the LOS was comparable between patients with and without sarcopenia, which is consistent with this present study [16,18]. In a recent meta-analysis, patients with sarcopenia had a higher 30-day mortality rate (odds ratio 2.38) [56]. This was not confirmed in this present study, where there was no mortality in patients with and without sarcopenia. Additionally, no significant effect of preoperative sarcopenia on the rate of postoperative complications was identified. In contrast, a recent meta-analysis that included 7176 patients that underwent gastrointestinal cancer surgery found that the preoperative incidence of sarcopenia was associated with elevated risks of both major and total complications [11]. Interestingly, in that study, a subgroup analysis was performed after stratifying studies by the use of ERAS care among patients with colorectal liver metastases and liver cancer. In that analysis, the risk ratio showed that sarcopenia did not significantly increase the risk of complications [11]. Moreover, that result suggested that ERAS care, which was provided for all patients in the present study, might have offered some compensation for sarcopenia. Indeed, ERAS care involves routine, dedicated preoperative counseling and education, perioperative nutrition management before liver surgery, and preconditioning prior to surgery. Thus, ERAS care may well reduce the risk associated with sarcopenia in patients undergoing surgery [57]. However, in view of the short preoperative period, it seems unlikely that the parameters of sarcopenia can be completely altered simply with nutritional interventions. It would also be necessary to intervene on physical condition, and over a longer preoperative period, without exposing the patients to the risk of oncological progression. Consequently, sarcopenia could be managed preoperatively, whereas many other risk factors, such as age, sex, or ASA score, are not modifiable or unlikely to improve during a short preoperative time period.

Sarcopenia can develop as a consequence of malignancy [45]. This association might explain our finding that malignancies were more common in the sarcopenia group. However, overall survival rates were similar between patients with and without preoperative sarcopenia. This finding was consistent with findings in several previous studies. In one study on 96 patients that underwent liver resection or liver transplantation for hepatocellular carcinoma (HCC), sarcopenia was not associated with long-term survival [18]. In another study, CT-based preoperative sarcopenia was assessed in 82 patients that were critically ill with cirrhosis, and sarcopenia had no prognostic value in predicting post-transplantation survival [58]. Similarly, in another study on 259 patients that underwent liver resections for colorectal metastases, sarcopenia was not significantly associated with recurrence-free or overall survival [17]. In contrast, a Japanese study showed that, in patients with HCC, preoperative sarcopenia was predictive of worse overall survival after a hepatectomy, even after adjusting for other known predictors [45]. Additionally, among patients that required liver transplants, sarcopenia was strongly correlated with post-liver transplantation mortality and 3-year survival [59]. Moreover, a meta-analysis that specifically studied patients with primary hepatic malignancies showed that patients with and without sarcopenia differed significantly in overall 1- and 3-year survival rates (1 year: OR: 0.43; *p* < 0.001; 3 years: OR: 0.67; *p* = 0.03) [15]. In the present study, the survival of patients with malignancy was less than 10% at 5 years, which appears to be low. However, studies of survival at 5 years after surgery remain rare and range between 10% and 30% at 5 years for cholangiocarcinoma, HCC, and colorectal metastases [60,61,62].

The differences among studies on the impact of sarcopenia on outcomes are probably due to multiple factors. Study results are likely to vary due to the different diagnostic modalities, and the different cut-off values used [18]. In addition, heterogeneity between study cohorts could lead to different findings on sarcopenia. In the present study, the cohort included patients that mainly underwent major hepatectomies for colorectal metastases, cholangiocarcinoma, and HCC. This heterogeneous cohort may have limited the ability to compare results to studies that focused on targeted cohorts. Furthermore, other factors probably influence oncological outcomes more than sarcopenia, such as tumor size, tumor grade, and the presence of vascular invasion, histological data, and systemic therapies. These co-founding factors were not considered in the survival analyzes in this present study.

This study has several limitations. Its single-center, retrospective design may have limited the ability to detect subtle differences between groups. The lack of a standardized preoperative CT imaging protocol and the variety of CT scanners and protocols used might have introduced heterogeneity in the imaging data set. These variations could have had small effects on the measurement of muscle quantity but mainly resulted in differences in the measurement of muscle quality, as previously reported [63]. However, this variety was equally distributed between the groups, and it reflected clinical practice settings. Patients with differences in diagnoses and demographics were included, which resulted in cohort heterogeneity. The different types of cases were well balanced between the two patient groups, but it may still have reduced the statistical power for detecting the effects of sarcopenia on outcomes. These differences were probably precisely related to the presence of sarcopenia, such as BMI, age, ASA score, and the presence of neoplasia, while cardiovascular and renal comorbidities, diabetes, and surgical details were comparable, for example. The cause-and-effect relationship is difficult to establish in the present study, and the correlation between sarcopenia and outcomes should therefore be interpreted with caution. The follow-up of clinical outcomes was limited to 30 postoperative days, according to the Clavien-Dindo classification, which can lead to an underestimation of complications and mortality. Oncological follow-up compliance was not measured; thus, survival rates may have been under- or overestimated. Many treatment modalities are available that are less invasive than surgery for unfit patients with hepatic pathologies. Therefore, it is possible that patients with minor frailties were prioritized for surgery, which could have introduced a selection bias. Patients with local extensive pathologies may also have been considered preferentially for surgery, which could potentially have led to a worse oncologic prognosis in comparison to locoregional treatments (chemoembolization, radiofrequency, or microwave, for example). Finally, although low muscle mass is a diagnostic criterion for sarcopenia, low muscle strength or physical performance, which are also required for the diagnosis of sarcopenia, were not measured [20,64]. The term “sarcopenia” might be considered a misnomer, even though low muscle mass is currently routinely used to define sarcopenia in the literature and in clinical practice. CT assessment of sarcopenia is relatively simple and economical because CT scans are routinely performed to support decisions regarding abdominal cancer surgery. However, there is a need for standardized cut-off values for assessing preoperative sarcopenia. Patients with gastric, esophageal, or pancreatic cancers that impact the gastrointestinal tract and cause feeding difficulties are more likely to be affected by sarcopenia, and these patients may benefit most from preoperative interventions [65,66]. More research is necessary for determining appropriate treatment modalities.

## 5. Conclusions

Based on CT assessment alone, preoperative sarcopenia has no impact on postoperative clinical outcomes or overall survival in patients that underwent liver resections. Future efforts should also consider muscle strength and physical performance, in addition to imaging, for preoperative risk stratification.

## Figures and Tables

**Table 1:** Demographics and surgical details of patients that underwent hepatectomies.

Item	Overall (*n* = 355)	Non-Sarcopenia (*n* = 143)	Sarcopenia (*n* = 212)	*p*-Value *
Age (years) (mean, SD)	62 (13.0)	59 (12.7)	64 (13.5)	<0.01
BMI (kg/m^2^) (mean, SD)	25.5 (4.5)	27.7 (4.9)	23.9 (3.5)	<0.01
Weight (kg) (mean, SD)	74 (15.3)	78 (16.4)	70 (13.7)	<0.01
Sex (male) (%)	210 (59.2)	71 (49.9)	139 (65.6)	<0.01
SMA (cm^2^) (mean, SD)	131.6 (32.7)	148.2 (34.7)	120.4 (25.8)	<0.01
SMRA (HU) (mean, SD)	39.5 (9.9)	40.2 (9.2)	39.0 (10.35)	0.090
IMATI (cm^2^/m^2^)	4.8 (3.1)	4.9 (3.5)	4.7 (2.7)	0.817
Cardiovascular disease (%)	16 (4.5)	5 (3.5)	11 (5.2)	0.604
Pulmonary disease (%)	27 (7.6)	10 (7.0)	17 (8.0)	0.839
Renal disease (%)	9 (2.5)	2 (1.4)	7 (3.3)	0.323
Diabetes (%)	39 (11.0)	17 (11.9)	22 (10.4)	0.730
ASA score ≥ 3 (%)	62 (17.4)	10 (7.0)	52 (24.5)	<0.01
Malignancy (%)	274 (77.2)	100 (69.9)	174 (82.5)	<0.01
Neoadjuvant treatment (%)	165 (46.5)	61 (42.7)	104 (49.1)	0.278
Diagnosis (%)				
Hepatocellular carcinoma	47 (13.2)	19 (13.3)	28 (13.2)	0.874
Colorectal metastases	155 (43.7)	51 (35.7)	104 (49.1)	0.016
Other metastases	23 (6.5)	10 (6.9)	13 (6.1)	0.657
Cholangiocarcinoma	55 (15.5)	22 (15.4)	33 (15.6)	1.000
Echinococcosis	39 (11.0)	25 (17.5)	14 (6.6)	<0.01
Other	36 (10.1)	16 (11.2)	20 (9.4)	0.587
Major hepatectomy (%)	190 (53.5)	73 (51.0)	117 (55.2)	0.450
Laparoscopy (%)	75 (21.1)	37 (25.9)	38 (17.9)	0.085

* Significant *p* values (<0.05) are displayed in bold characters. SD: standard deviation; BMI: body mass index; SMA: skeletal muscle area; SMRA: skeletal muscle radiation attenuation; HU: Hounsfield unit; IMATI: intramuscular adipose tissue index; ASA: American Society of Anesthesiologists.

**Table 2:** Clinical outcomes of patients that underwent hepatectomies.

Outcome	Overall (*n* = 355)	Non-Sarcopenia (*n* = 143)	Sarcopenia (*n* = 212)	*p*-Value
Length of stay (median, IQR)	8 (6–14)	8 (6–16)	8 (6–14)	0.753
30-day complications (%)				
Any	167 (47.0)	69 (48.3)	98 (46.2)	0.745
Major	40 (11.3)	16 (11.2)	24 (11.3)	1.000
Bile leakage (%)				0.156
A	12 (3.4)	9 (6.3)	3 (1.4)	
B	28 (7.9)	15 (10.6)	13 (6.2)	
C	5 (1.4)	1 (0.7)	4 (1.9)	
Liver failure (%)				0.203
A	2 (0.6)	-	2 (0.9)	
B	4 (1.2)	1 (0.7)	3 (1.4)	
C	1 (0.3)	-	1 (0.5)	
30-day mortality (%)	0 (-)	0 (-)	0 (-)	1.000
30-day reoperation (%)	29 (8.2)	9 (6.3)	20 (9.4)	0.328

IQR: interquartile range.

**Table 3:** Predictive factors of major complications.

Item	Univariate Analysis		Multivariate Analysis	
	OR (95% CI)	*p*-Value	OR (95% CI)	*p*-Value
Age	1.030 (1.001–1.059)	0.046 *	1.027 (0.997–1.058)	0.073
Sex	1.498 (0.745–3.012)	0.257	-	-
BMI	1.012 (0.942–1.087)	0.746	-	-
ASA score	1.357 (0.494–3.725)	0.553	-	-
SMA	1.003 (9.993–1.013)	0.590	-	-
SMI	1.004 (0.969–1.040)	0.836	-	-
SMRA	0.974 (0.942–1.008)	0.974	-	-
IMATI	0.984 (0.881–1.099)	0.776	-	-
Hepatocellular carcinoma	0.513 (0.151–1.737)	0.283	-	-
Colorectal metastases	0.450 (0.217–0.933)	0.032 *	0.627 (0.277–1.422)	0.264
Cholangiocarcinoma	3.702 (1.785–7.678)	<0.001 *	2.762 (1.218–6.264)	0.015 *
Echinococcosis	1.181 (0.433–3.217)	0.745	-	-

* Significant *p* values (<0.05); BMI: body mass index; ASA: American Society of Anesthesiologists; SMA: skeletal muscle area; SMI: skeletal muscle index; SMRA: skeletal muscle radiation attenuation; IMATI: intramuscular adipose tissue index.

## Data Availability

The data that support the findings of this study are available from the corresponding author upon reasonable request.

## Supplementary Materials

The following supporting information can be downloaded at: https://www.mdpi.com/article/10.3390/cancers14010261/s1, Table S1: Clinical outcomes of patients that underwent major hepatectomies.
