# Supplementary material for: Association between CT-Based Preoperative Sarcopenia and Outcomes in Patients That Underwent Liver Resections
Source: Cancers (Basel). 2022 Jan 5;14(1):261. doi: 10.3390/cancers14010261 (PMC8750804; doi:10.3390/cancers14010261)
Supplement: Supplementary file 1 [file cancers-14-00261-s001.zip › cancers-1454957-supplementary.pdf]

cancers MDPI

Supplementary Files

Associations between CT-Based Preoperative Sarcopenia and Outcomes in Patients That Underwent Liver Resections

David Martin, Yaël Maeder, Kosuke Kobayashi, Michael Schneider, Joachim Koerfer, Emmanuel Melloul, Nermin Halkic, Martin Hübner, Nicolas Demartines, Fabio Becce and Emilie Uldry

Table S1. Clinical outcomes of patients that underwent major hepatectomies

Outcome	Overall (*n* = 190)	Non-sarcopenia (*n* = 73)	Sarcopenia (*n* = 117)	*p*-value
**Length of stay** (median, IQR)	12 (7-20)	11 (7–19)	13 (8–21)	0.196
**30-day complications,** (%)				
Any	118 (51)	51 (70)	67 (57)	0.092
Major	31 (16)	12 (16)	19 (16)	1.000
**Bile leakage**, (%)				0.122
A	9 (5)	7 (10)	2 (2)	
B	26 (14)	14 (19)	12 (10)	
C	3 (2)	-	3 (3)	0.532
**Liver failure**, (%)				
A	2 (1)	-	2 (2)	
B	4 (2)	1 (1)	3 (3)	
C	1 (1)	-	1 (1)	
**30-day reoperation**, (%)	24 (13)	6 (8)	16 (114)	0.352

IQR: interquartile range.
